# Supplementary figures and images for: Identification of ligand and receptor interactions in CKD and MASH through the integration of single cell and spatial transcriptomics
Source: PLoS One. 2024 May 20;19(5):e0302853. doi: 10.1371/journal.pone.0302853 (PMC11104622; doi:10.1371/journal.pone.0302853)

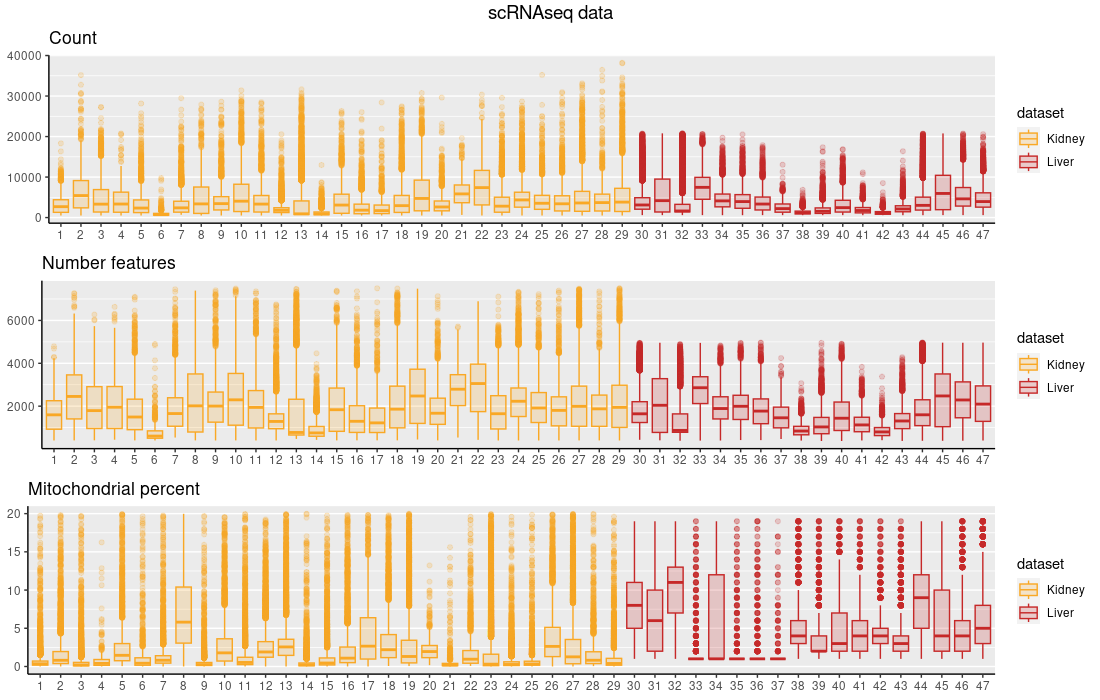

Supplement: S1 Fig — (TIF) [file pone.0302853.s007.tif]

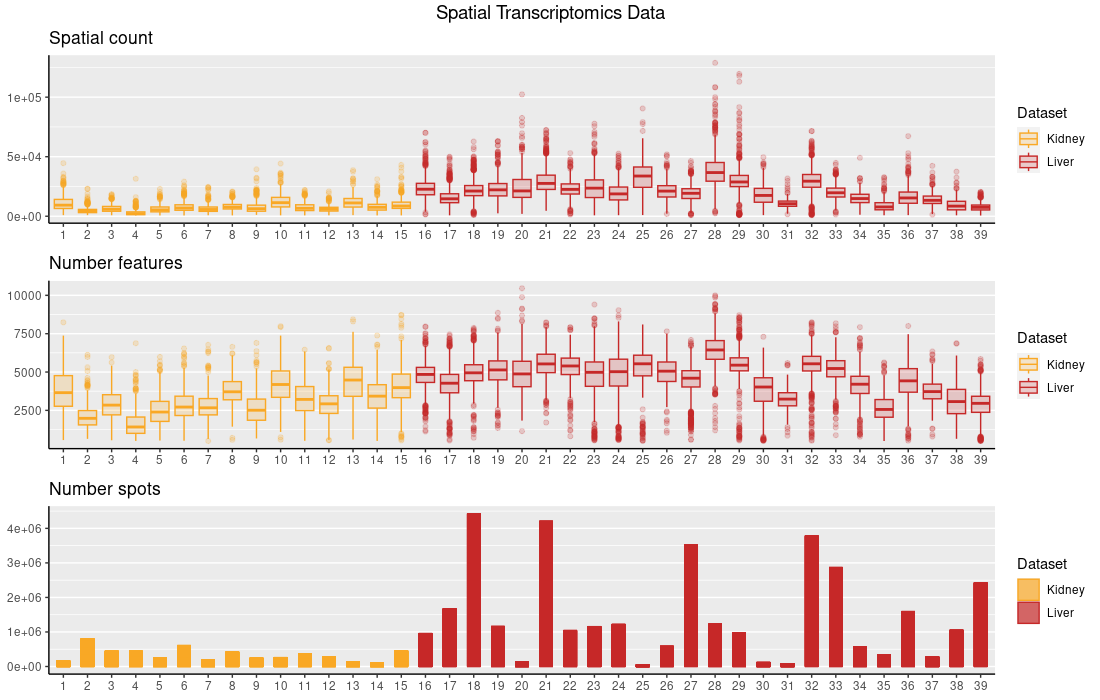

Supplement: S2 Fig — (TIF) [file pone.0302853.s008.tif]

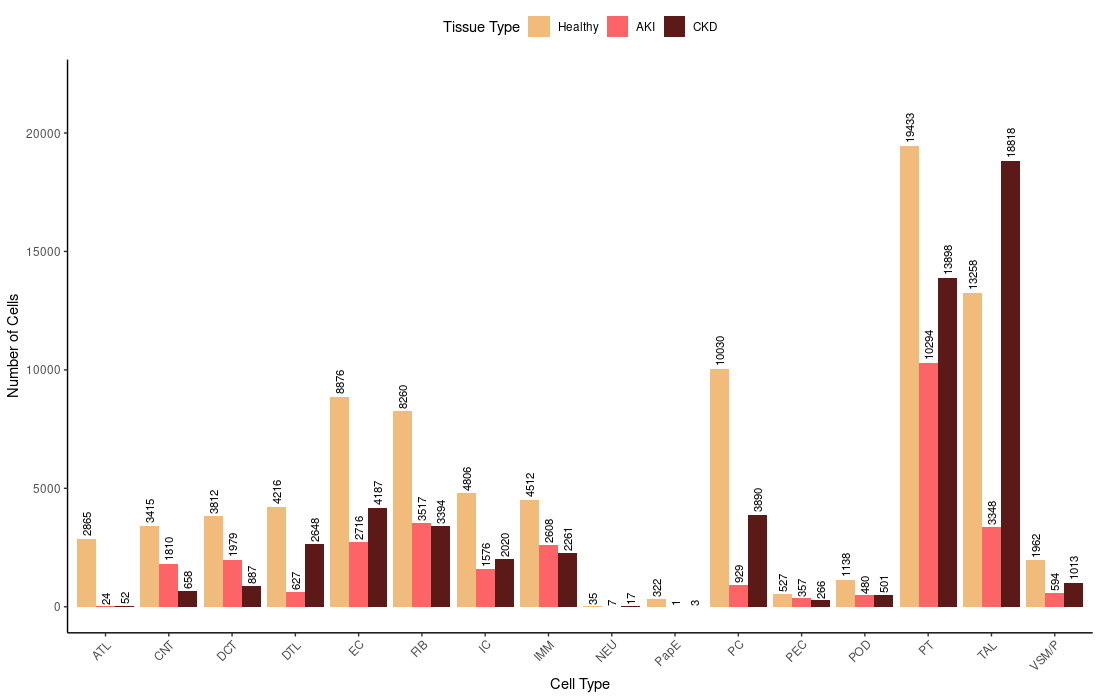

Supplement: S3 Fig — (TIF) [file pone.0302853.s009.tif]

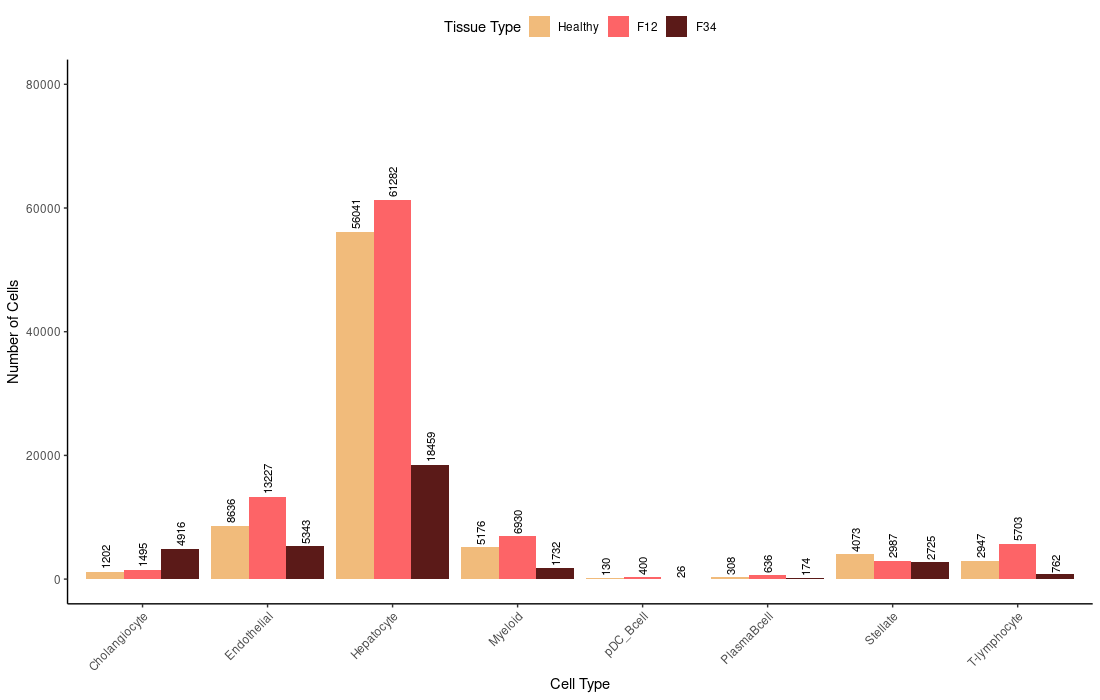

Supplement: S4 Fig — (TIF) [file pone.0302853.s010.tif]

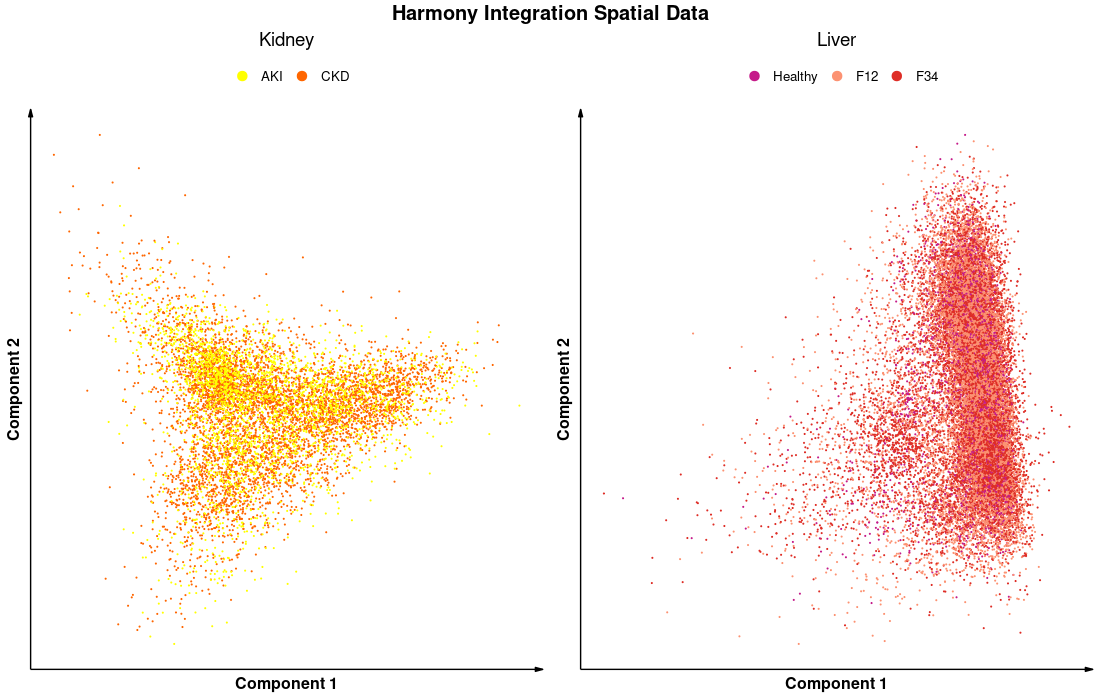

Supplement: S5 Fig — (TIF) [file pone.0302853.s011.tif]

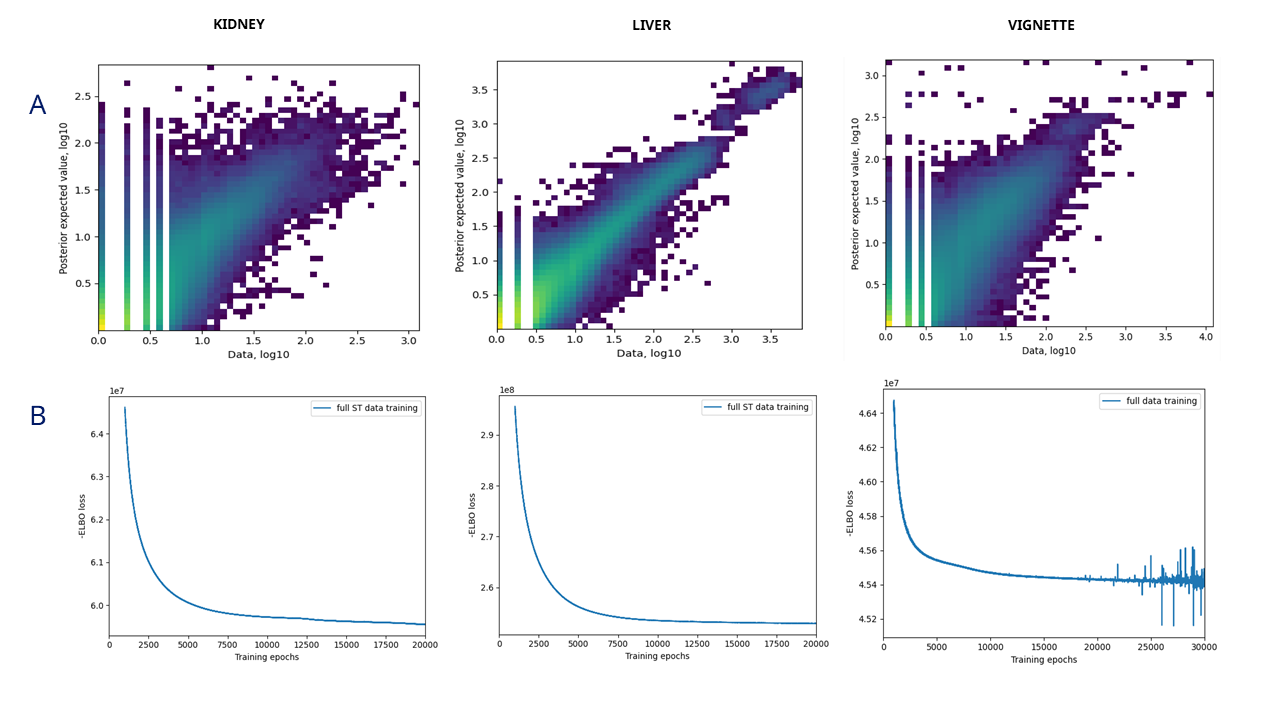

Supplement: S6 Fig — A. 2D histogram showing the reconstruction accuracy. B. ELBO loss history during training deconvoluting the spatial datasets. (TIF) [file pone.0302853.s012.tif]

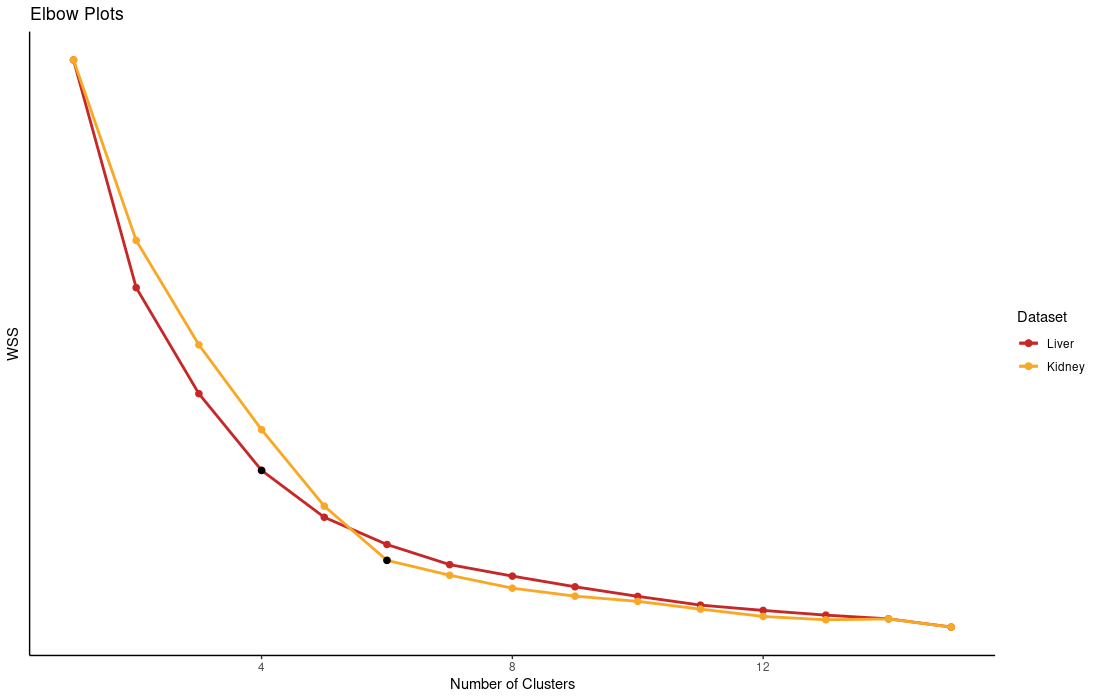

Supplement: S7 Fig — (TIF) [file pone.0302853.s013.tif]

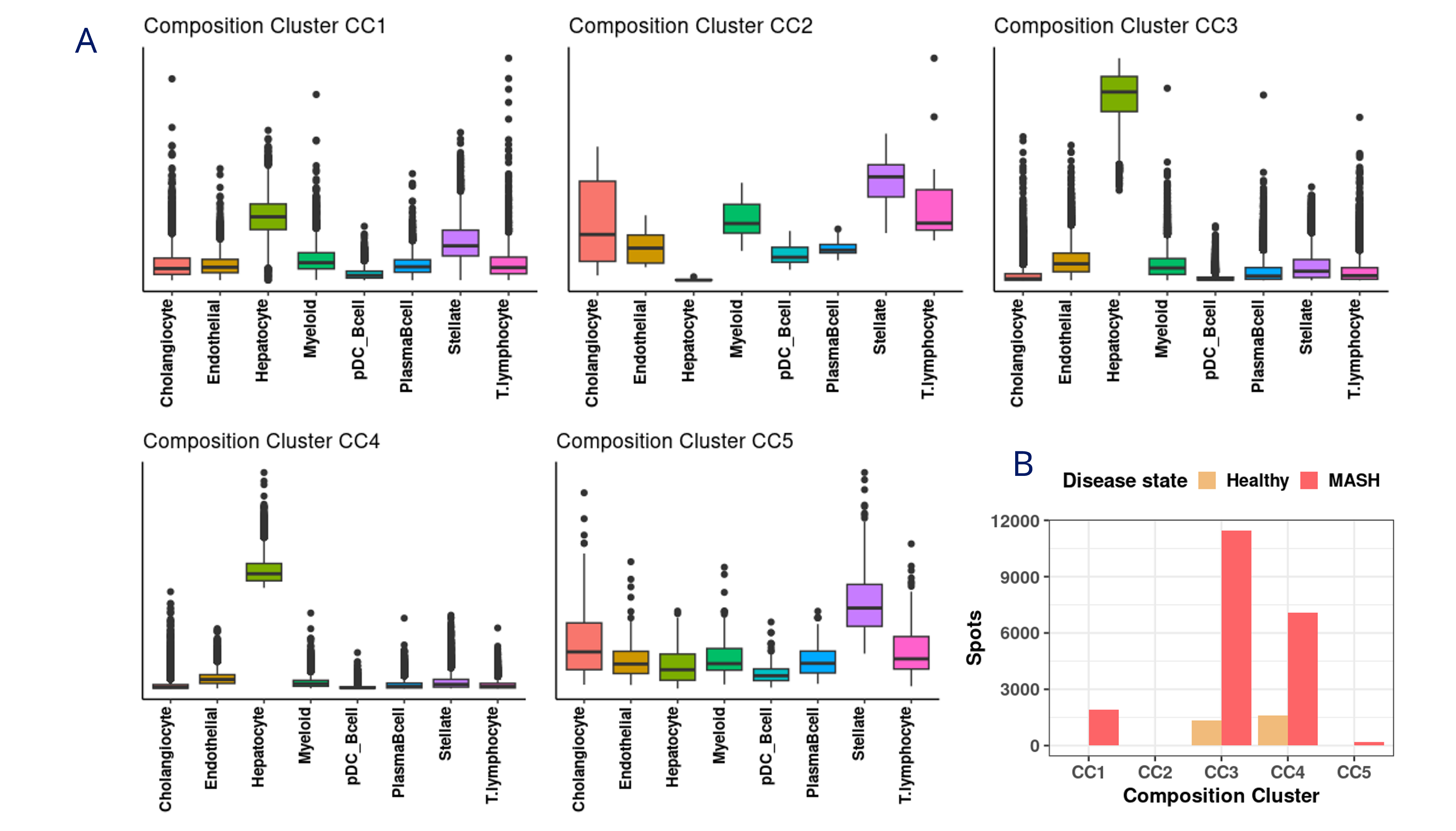

Supplement: S8 Fig — A. Composition clusters of the cell deconvolution results in spatial data. B. Contribution of the spots to each disease status per composition cluster. (TIF) [file pone.0302853.s014.tif]

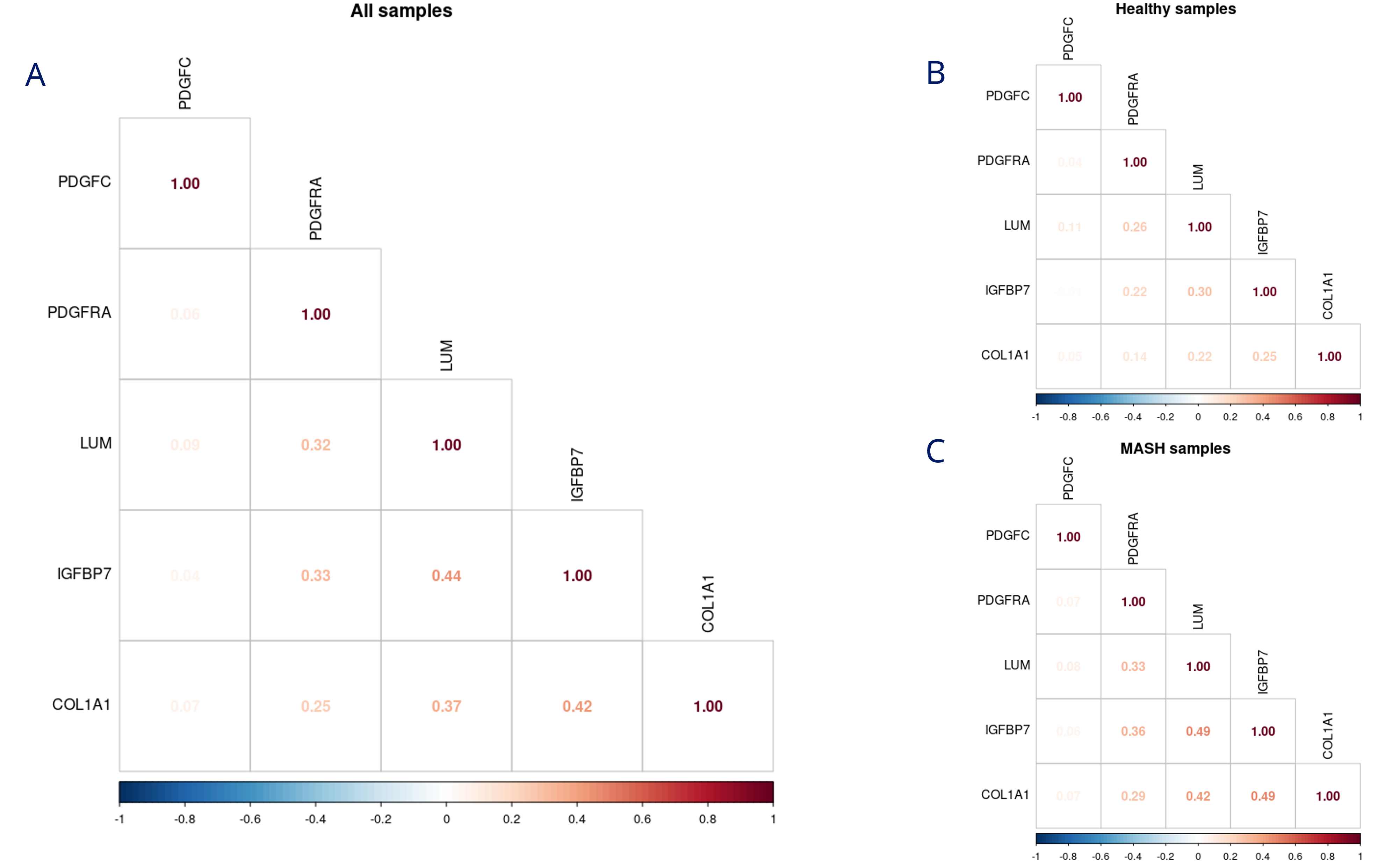

Supplement: S9 Fig — A. Correlation considering all the samples. B. Correlation considering only Healthy samples. C. Correlation considering only MASH samples. (TIF) [file pone.0302853.s015.tif]

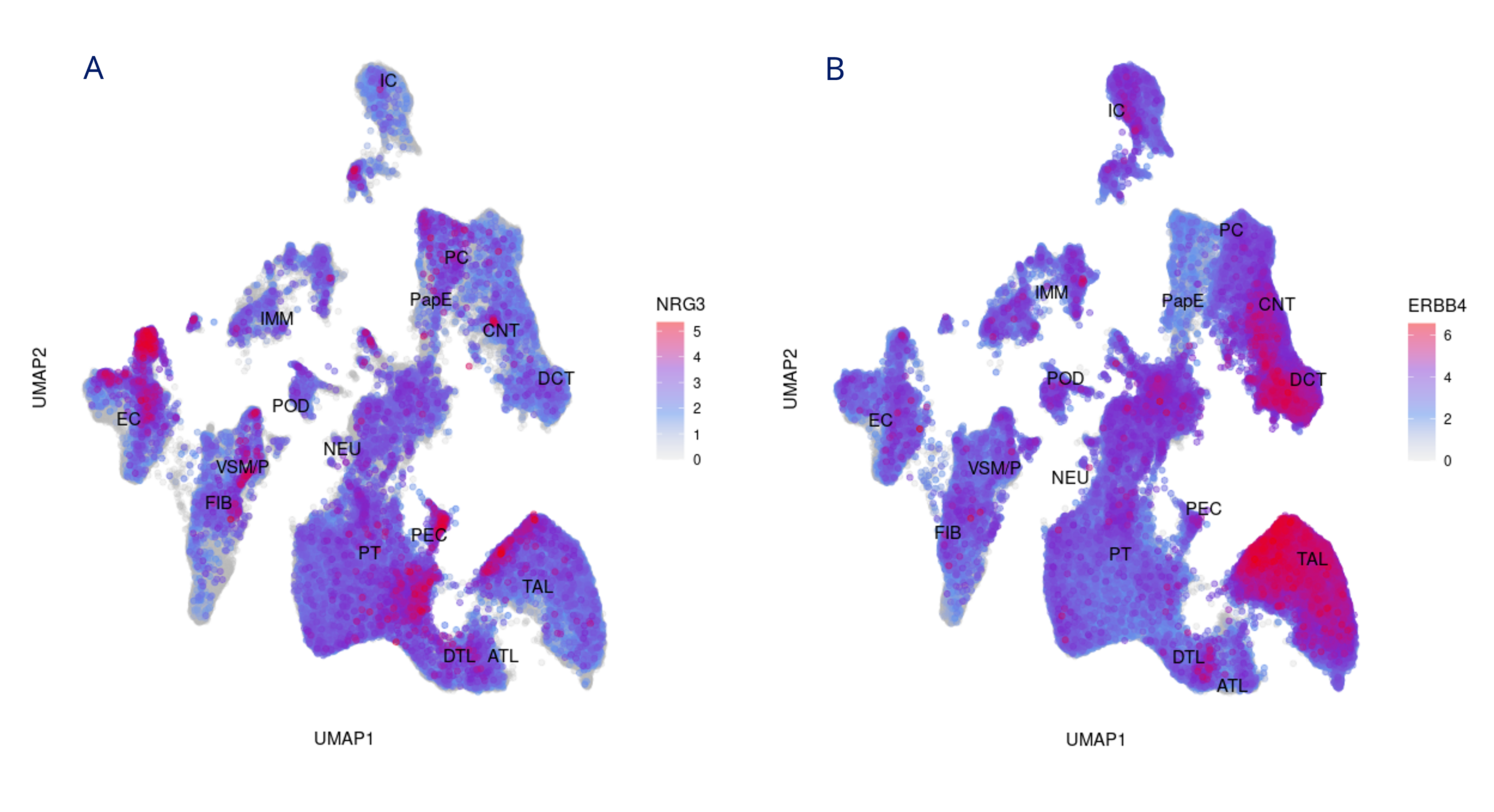

Supplement: S10 Fig — (TIF) [file pone.0302853.s016.tif]
