# Supplementary material for: Identification of ligand and receptor interactions in CKD and MASH through the integration of single cell and spatial transcriptomics
Source: PLoS One. 2024 May 20;19(5):e0302853. doi: 10.1371/journal.pone.0302853 (PMC11104622; doi:10.1371/journal.pone.0302853)
Supplement: Attachment — Submitted filename: Response to Reviewers.docx [file pone.0302853.s018.docx]

# Response to Reviewers

## Journal requirements.

**In line 96, there should be total 12 samples (3 healthy individuals and 9 patients with MASH).**

Number of samples are now fixed.

**In the Table S2, the numbers should be corrected accordingly. Specifically, "F1-F2" should be "7" instead of "13". "MESH" total of F1-F2 and F3-F4 should be "9" instead of "15". The title of the table indicates" 1 Two samples did not fulfill the criteria for MASH". So please label "1" in the table.**

Number of samples in the Table S2 has been updated. Label “1” has been replaced with “*” to ease formatting in the excel file.

**In the Table S3, "Unknown" "ScRNAseq" should be "9" instead of "15".**

Table S2 has been now fixed.

**Please review your reference list to ensure that it is complete and correct. If you have cited papers that have been retracted, please include the rationale for doing so in the manuscript text, or remove these references and replace them with relevant current references. Any changes to the reference list should be mentioned in the rebuttal letter that accompanies your revised manuscript. If you need to cite a retracted article, indicate the article’s retracted status in the References list and include a citation and full reference for the retraction notice. Line 619, Ref 41 shows "Invalid citation".**

A problem with Endnote was causing the invalid citation, it has now been fixed.
